# Modified step aerobics training and neuromuscular function in osteoporotic patients: a randomized controlled pilot study

**DOI:** 10.1007/s00402-016-2607-5

**Published:** 2016-12-16

**Authors:** Martin Behrens, Karoline Müller, Jill-Isabel Kilb, Lennart Schleese, Philipp K. E. Herlyn, Sven Bruhn, Thomas Mittlmeier, Hans-Christof Schober, Dagmar-C. Fischer

**Affiliations:** 1 Institute of Sport Science, University of Rostock, Rostock, Germany; 2 Department of Traumatology, Hand- and Reconstructive Surgery, University Medicine Rostock, Rostock, Germany; 3 Department of Internal Medicine, Klinikum Südstadt, Rostock, Germany; 4 Department of Pediatrics, University Medicine Rostock, Ernst-Heydemann-Str. 8, 18057 Rostock, Germany

**Keywords:** Aging, Maximal voluntary contraction strength, H-reflex, V-wave

## Abstract

**Background:**

Training programs directed to improve neuromuscular and musculoskeletal function of the legs are scarce with respect to older osteoporotic patients. We hypothesized that a modified step aerobics training program might be suitable for this purpose and performed a randomized controlled pilot study to assess the feasibility of conducting a large study. Here we report on the training-related effects on neuromuscular function of the plantar flexors.

**Patients and methods:**

Twenty-seven patients with an age of at least 65 years were enrolled and randomized into control and intervention group. The latter received supervised modified step aerobics training (twice weekly, 1 h per session) over a period of 6 months. At baseline, and after 3 and 6 months neuromuscular function of the plantar flexors, i.e., isometric maximum voluntary torque, rate of torque development and twitch torque parameters were determined in detail in all patients of both groups.

**Results:**

Twenty-seven patients (median age 75 years; range 66–84 years) were randomized (control group *n* = 14; intervention group *n* = 13). After 3 and 6 months of training, maximum voluntary contraction strength in the intervention group was significantly higher by 7.7 Nm (9.1%; 95% CI 3.3–12.2 Nm, *P* < 0.01) and 12.4 Nm (14.8%; 95% CI 6.4–18.5 Nm, *P* < 0.01) compared to controls. These changes were most probably due to neural and muscular adaptations.

**Conclusion:**

It is worthwhile to investigate efficacy of this training program in a large randomized trial. However, a detailed neuromuscular assessment appears feasible only in a subset of participants.

## Background

Aging is associated with a decline in muscle strength (dynapenia) caused by the loss of muscle mass, impairments of intrinsic force-generating properties of skeletal muscles and changes in the structure and function of the nervous system [1]. These processes are paralleled by a loss of bone mineral density (BMD) and strength (for reference see [2, 3] and literature cited therein). Thus, in humans over the age of 65 years the age-related deteriorations of the musculoskeletal unit and physical capabilities are significantly associated with an increased risk of falls [4, 5]. The latter is especially relevant in patients with osteoporosis. In these patients even low-energy traumata are associated with an increased risk of fractures with concomitantly high morbidity and mortality [6–8].

Physical activity has been shown to improve neuromuscular and musculoskeletal functions in osteopenia and osteoporotic patients [9–12]. These effects are strongly related to the type of exercise, i.e., dynamic exercises including appropriate mechanical stress are required to stimulate strength of the musculoskeletal unit (for review see [13, 14]). The development of adequate training programs for older patients is especially challenging. This might be due to physical disabilities, such as poor postural control and overall frailty. Furthermore, cognitive impairments and/or intellectual disabilities have to be taken into account. Improvement or at least preservation of bipedal locomotion and postural control require to target neuromuscular and musculoskeletal function of the legs.

Stair climbing is important for independent living and an impact exercise, as well. In fact, ground reaction forces during ascending and descending stairs are proportional to approximately 1.3 times the body weight [15]. We suppose that step aerobics training might be suitable for these purposes even in older patients with osteoporosis, and worthwhile to be investigated in a large randomized trial. There are few reports on the effects of step aerobics training in older patients at risk for the development of osteoporosis [16–19]. Furthermore, the design and results of a community-based randomized trial conducted in Australia were reported recently [20, 21]. Although step aerobics training was introduced about 30 years ago, it appears questionable to us whether this type of intervention will be accepted by older patients with osteoporosis in this part of eastern Germany. Especially, older patients who know about the consequences of osteoporosis and/or the sequelae associated with fractures might be anxious to perform this type of training. Thus, to conduct a sufficiently powered large trial on this issue is challenging regarding the age of the target group and the type of intervention. Therefore, we decided to do a randomized controlled pilot study first to gain information on the specific requirements for a large clinical trial. Beyond this, we investigated neuromuscular function of the plantar flexors in detail, as these are not only thought to be improved by this form of training but are also important for stair climbing and independent living.

## Materials and methods

### Study protocol

**Fig. 1:**
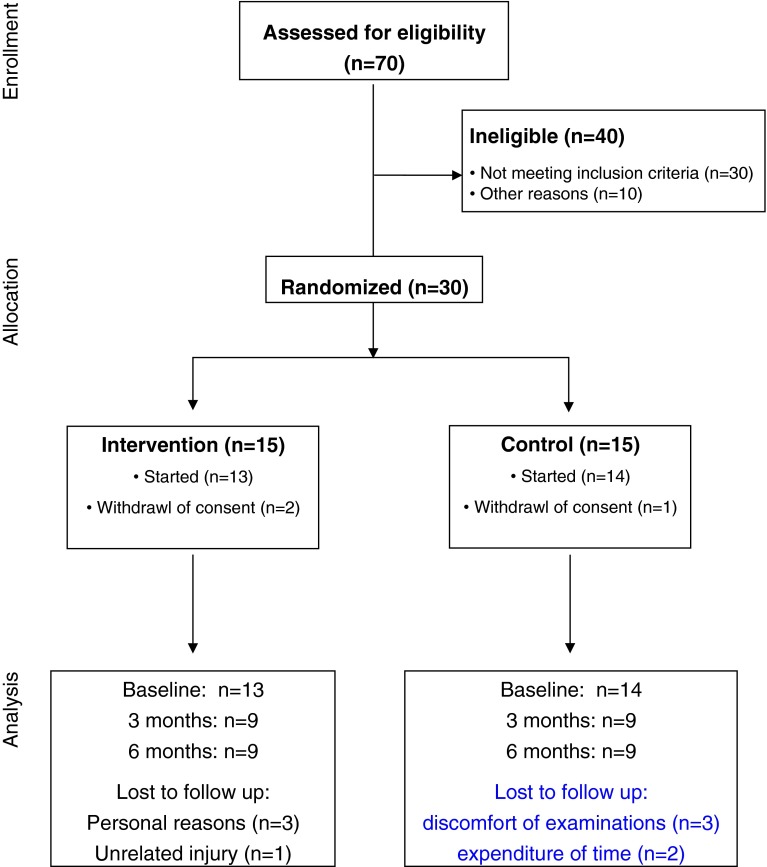
Flow of the participants from recruitment to study examination

This was a randomized controlled 6-months pilot study directed to gain information on the specific requirements for a large clinical trial on a modified step aerobics training program as an intervention in older patients with osteoporosis. Neuromuscular function was thoroughly assessed at baseline and throughout the study period to quantify training-related effects (proof-of-concept). The study was conducted according to the declaration of Helsinki (Version 2008), was approved by the university’s ethics committee August 2012 and retrospectively registered at the German Clinical Trial Register January 2016. All participants gave written informed consent and the flow of patients is given in Fig. 1. We announced the study in our outpatient clinic by a flyer, referred to it during counseling (HCS, TM). In addition, we identified suitable patients by chart review and invited them personally. Several open oral presentations were given to the potential collective of candidates to introduce our concept in detail.

### Patients

*Inclusion criteria*: age ≥ 65 years, and clinically diagnosed osteoporosis, i.e., a T-score ≤ −2.5 standard deviation at the lumbar spine and/or femoral neck (determined by DXA at max. 36 months prior to enrolment) and/or a history of osteoporotic fractures, i.e., a fracture of either radius, humerus, vertebra or femur secondary to a fall (low-energy trauma). Additional criteria were walking speed ≥ 0.8 m·s^−1^, chair rising test ≤ 10 s, body mass index ≥ 20 kg/m^2^, and at least one self-reported fall during the last 12 months. *Exclusion criteria*: active participation in a strength and/or endurance training during a 24-months period prior to enrolment, sports activity ≥ two times a week, cardiac insufficiency, cardiac pacemaker, chronic renal failure, non-curative treated tumors, systemic application of corticosteroids, peripheral arterial occlusive disease, poor mobility, i.e., walking more than 5 min not possible, severe osteoarthritis, rheumatism and/or severely impaired balance, planned absence of more than two weeks during the study period.

Clinical data on prescribed medications and history of disease were obtained by interview and chart review, respectively.

Group assignment (block randomization) was carried out using sealed, opaque envelopes with consecutive numbering and stratification according to sex and age. The person who opened the envelopes and carried out the group assignment was not involved in the generation and allocation concealment.

### Study intervention

At baseline (t_0_) as well as 3 and 6 months (t_1_, t_2_) after study onset, the performance capacity at the anaerobic threshold was determined by means of an incremental bicycle ergometer test [22]. Blood lactate was measured at each step, whereas heart rate was monitored continuously (Acentas, Germany) during the entire ergometry. From these data the anaerobic threshold was calculated. Continuous individual online heart rate monitoring (Acentas, Germany) was performed throughout the training sessions to keep the intensity at the anaerobic threshold during the loading phase and to ensure that the heart rate was below 90 bpm during the resting phase.

The intervention group trained twice a week for 6 months at a local sports club. Each session was supervised by more than one experienced instructor (KM, JIK, LS, AS, and RD). The exercises (modified step aerobics) were designed to increase strength, balance and agility. The patients were familiarized with a sequence of steps and movements (choreography) during the first 4 weeks. Each training session (60 min) started with a 10 min warm-up period consisting of basic aerobic steps without using the stepper, followed by step aerobics (30 min), balance training (10 min) and finally a 10 min cool-down period. The physical activities were accompanied by music. The patients danced a sequence of turns, forward, backward and side stepping with concomitant use of the arms and requirement of the stepper. For balance training the patients performed a sequence of parallel, tandem and one-legged stances with and without retroversion of the other leg and with eyes open and closed, respectively. During cool-down stretching and breathing exercises were performed. The control group was equipped with a step counter and was asked to maintain their individual level of physical activities.

### Study examinations

Routine laboratory work-up prior at baseline was according to current guidelines and included alkaline phosphatase, parathyroid hormone, 25-hydroxyvitamin D_3_, sex hormones, creatinine, calcium and phosphate [23]. Blood samples were analyzed with established laboratory procedures. Acceptance of the intervention and the osteoporosis-related quality of life was assessed by interview and questionnaires, respectively [24, 25]. Neuromuscular function was investigated at baseline and after 3 months and 6 months of training. All study examinations were done on days without exercise. Participants were asked to refrain from consuming alcohol and caffeine and not to perform any strenuous exercise the day before the measurement of neuromuscular function.

### Assessment of neuromuscular function

**Fig. 2:**
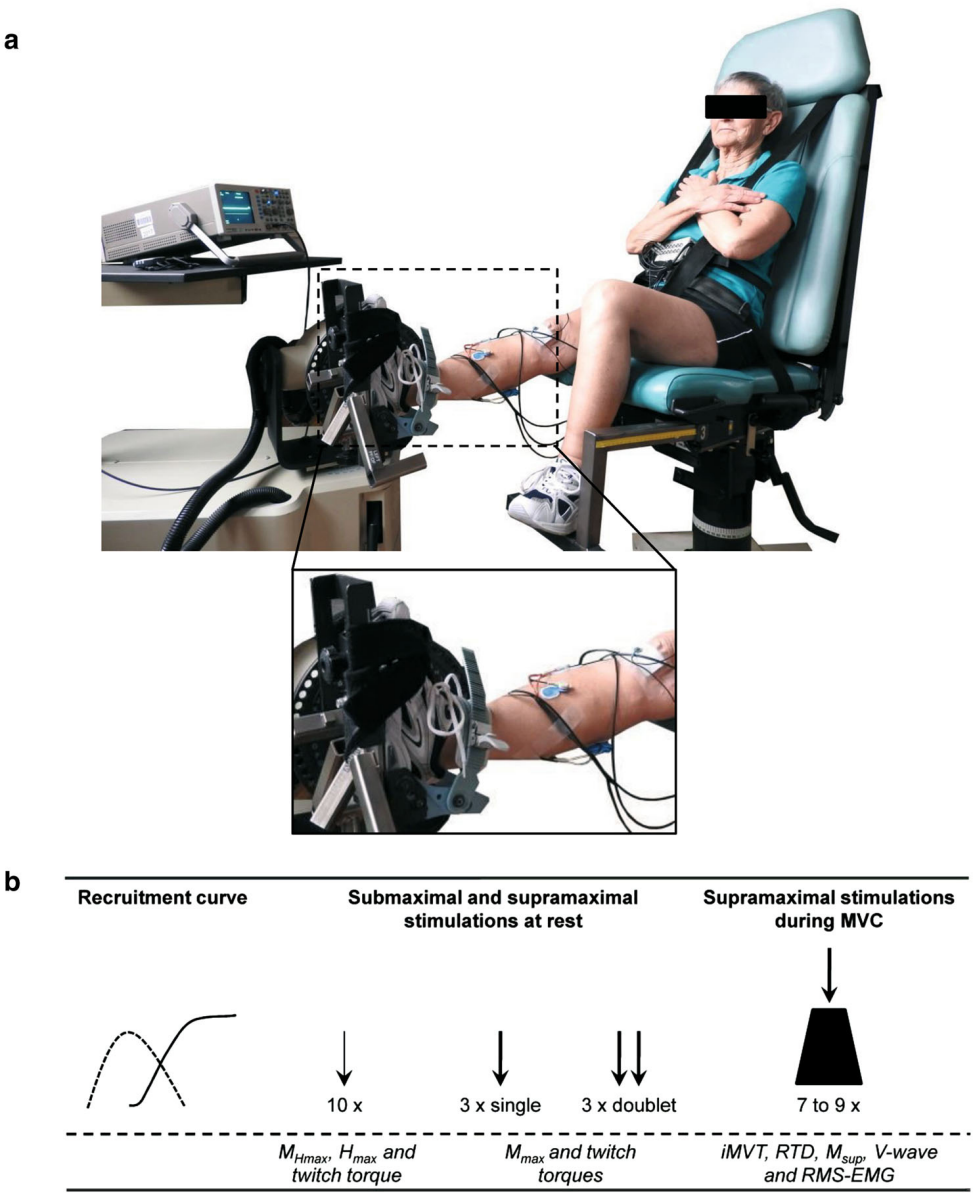
A patient placed for examination of neuromuscular function (**a**) with a schematic overview of the procedures carried out during neuromuscular testing and the parameters extracted (**b**). **a** An image of a patient sitting in the dynamometer. The enlarged picture shows the foot attached to the adapter, the EMG electrodes (*blue*) and the anode distal to the patella (*white*) used for electrical stimulation of the posterior tibial nerve. **b** The procedures carried out during neuromuscular testing and the parameters extracted. The *thin arrow* indicates stimulation at *H*_max_ intensity and the *thick arrow* indicates stimulation at supramaximal intensity. *M*_*Hmax*_ submaximal M-wave evoked at *H*_max_ intensity, *H*_*max*_ maximal H-reflex, *M*_*max*_ maximal M-wave, *M*_*sup*_ maximal M-wave during MVC, *iMVT* isometric maximum voluntary torque, *RTD* rate of torque development, *RMS-EMG* root mean square of the EMG signal

The patients were comfortably seated in a standardized position on a CYBEX NORM dynamometer (Computer Sports Medicine^®^, Inc., Stoughton, MA). Measurements were made on the triceps surae muscle of the right leg. Although the quadriceps muscles are equally relevant in this group of patients, we chose the triceps surae because stimulation of the posterior tibial nerve is better tolerated than stimulation of the femoral nerve. Before neuromuscular testing, the patients sat passively on the dynamometer for ~5 min to rule out any effects from walking to the laboratory. Neuromuscular function of the plantar flexors was assessed under the same standardized conditions and without a preceding warm-up period to avoid H-reflex and M-wave potentiation [26]. The posterior tibial nerve was submaximally and supramaximally stimulated at rest and during maximum voluntary contraction (MVC) (Fig. 2).

### Electrical stimulation

Transcutaneous electrical stimulation of the posterior tibial nerve in the popliteal fossa was used to generate the stimulus–response curves as described previously [27].

Briefly, the posterior tibial nerve was stimulated using a self-adhesive electrode (1 cm diameter). The anode was a self-adhesive electrode (40 × 90 mm, Spes Medica, Italy) fixed immediately distal to the patella on the anterior aspect of the knee. It was ensured that the electrical stimulation did not activate the tibialis anterior muscle. The percutaneous electrical stimuli were single pulses (1 ms duration, 400 V maximal voltage) delivered by a constant-current stimulator (Digitimer^®^ DS7A, Hertfordshire, UK). The inter stimulus intervals (ISI) were provided by a Digitimer^®^ train/delay generator (DG2A, Hertfordshire, UK). The testing procedure included random stimulation (ISI 7 s) with different current intensities, resulting in a recruitment curve. During this procedure, peak-to-peak maximal H-reflex (H_max_) and maximal M-wave (M_max_) of the soleus muscle were determined. M_max_ responses at rest and during MVC (M_sup_) as well as V-wave responses during MVC were evoked with supramaximal stimulation intensity (140%).

Resting twitch torques were evoked using supramaximal single (1 ms duration, 400 V maximal voltage) and doublet stimuli (1 ms duration, 10 ms apart, 400 V maximal voltage). To normalize muscle activity of the tibialis anterior muscle, M_max_ amplitudes were evoked three times by stimulating the peroneal nerve close to the fibular head with supramaximal stimuli.

### EMG and torque recordings

Surface EMG was recorded using bipolar EMG Ambu^®^ Blue Sensor N electrodes (2 cm diameter) as described previously [27]. The electrodes were firmly attached to the shaved, abraded and cleaned skin over soleus, medial gastrocnemius, lateral gastrocnemius and tibialis anterior of the right leg. The reference electrode was attached to the tibia of the ipsilateral leg. Signals were amplified (2500×), band-pass filtered (10-450 Hz) and digitized with a sampling frequency of 5 kHz through an analog-to-digital converter (DAQ Card™-6024E, National Instruments, USA). Both, the EMG and torque signals were sampled at 5 kHz and stored on a hard drive for later analysis with a custom built LABVIEW^®^ based program (Imago, Pfitec, Germany).

Torque signals as the result of muscle contraction were measured using a CYBEX NORM dynamometer (Computer Sports Medicine^®^, Inc., Stoughton, MA). The individual positioning for each patient was similar throughout the examinations, i.e., they were seated with their knees straight and their feet firmly attached to the adapter of the dynamometer [27, 28] (Fig. 2). For this purpose, velcro straps and a snowboard binding were used and all participants wore shoes provided by the laboratory to guarantee the same material properties throughout all measurements. An ankle joint angle and hip joint angle of 90° and 80°, respectively (0° = full extension), were maintained during the sessions. The participants` ankle joints were aligned with the axis of the dynamometer. Straps across the thigh, waist and chest prevented excessive movements. Isometric maximum voluntary torque (iMVT) generation, i.e., the result of MVC, was tested by asking the participants to exert isometric maximal voluntary plantar flexions against the metal plate of the dynamometer for 3 s. For each trial, participants were thoroughly instructed to act as forcefully and as fast as possible. They were motivated by strong verbal encouragement and online visual feedback of the instantaneous dynamometer torque provided on a digital oscilloscope (HM1508, HAMEG Instruments, Germany). Care was taken that the MVC trials were performed without apparent countermovement or pre-tension (change of baseline torque <0.5 Nm during the 200 ms prior to contraction onset). A rest period of 1 min was allowed between the trials. Before MVC testing, the participants performed three to five MVC familiarization trials. When the coefficient of variance of three subsequent trials was below 5%, MVC testing was started. The participants performed seven to nine isometric maximal voluntary plantar flexions for V-wave testing. It has been shown that eight MVC attempts are sufficient to get reliable results for the evoked potentials [29].

### Data analysis

The torque signals were corrected for the effect of gravity. The mean of the respective signals recorded during the MVC trials was used to calculate rate of torque development (RTD), iMVT and muscle activity. Explosive voluntary strength was determined by calculating the average RTD over time intervals of 0–50 and 0–200 ms relative to the onset of contraction.

Muscle activation during the early phase of contraction was analyzed by calculating the root mean square of the amplitude of the EMG signal (RMS-EMG) over time intervals of 0–50 and 0–200 ms relative to the onset of the EMG signals. RMS-EMG during iMVT (RMS-EMG_iMVT_) was calculated over a 200 ms period at iMVT, i.e., 200 ms prior to the electrical stimulus. To reduce errors due to electrode relocation and between-session variability in skin impedance, subcutaneous fat and fascia muscle activity was normalized, i.e., RMS-EMG of soleus, medial gastrocnemius, lateral gastrocnemius and tibialis anterior were divided by their respective *M*_max_ values (RMS-EMG/*M*_max_). Furthermore, RMS-EMG/*M*_max_ was averaged across soleus, medial gastrocnemius, and lateral gastrocnemius to calculate triceps surae activation during the early phase of contraction and at iMVT (RMS-EMG_RTD_/*M*_max_ and RMS-EMG_iMVT_/*M*_max_, respectively). Torque and EMG onsets were identified manually according to the method of Tillin et al. [30].

*H*_max_, *M*_Hmax_, *M*_max_, *M*_sup_ and V-wave amplitudes were measured peak-to-peak and averaged, respectively. The *H*_max_/*M*_max_-ratio was used as a global index of modulations at the spinal level due to alterations in α-motoneuron excitability and/or presynaptic inhibition of primary muscle spindle afferents [31]. The *M*_Hmax_/*M*_max_-ratio was determined to ensure that the same proportion of α-motoneurons was activated by the electrical stimulation. The *V*/*M*_sup_-ratio reflects the neural drive from spinal α-motoneurons to the soleus muscle [32]. Resting twitch torques were averaged and analyzed regarding their peak twitch torque, i.e., the highest value of twitch torque signal [27].

### Outcome measure

Recruitment rates, feasibility of inclusion-/exclusion criteria, the acceptance of the intervention (intensity per training session, frequency of training) and quality of life were evaluated ([24, 25]. The changes in neuromuscular function were used as preliminary estimates of effectiveness (proof-of-concept).

### Statistical analysis

Data were checked for normal distribution using the Shapiro–Wilk test. The statistical approach comprised the analysis of covariance (ANCOVA) with baseline measurement and sex entered as covariates [33]. SPSS 20.0 (SPSS Inc., Chicago, IL, USA) was used for statistical analysis. Data obtained at baseline are presented as mean ± standard deviation, those obtained after 3 and 6 months of training are given as baseline-adjusted means ± baseline-adjusted standard deviation. If appropriate, data are presented as difference between means (95% confidence interval). All *P* values are two-sided and a *P* value below 0.05 was considered significant.

## Results

### Recruitment and patient characteristics

Patients were recruited during a three months period (late summer to autumn 2012), baseline examinations (4–6 participants per day) were performed during November 2012 and patients were randomized subsequently. The intervention was between December 2012 and June 2013. Individual study examinations were scheduled according to the protocol. Roughly, half of the participants from either group extended the training under the same supervisors for additional 6 months, although without concomitant examinations and/or follow-up examinations. The flow of participants through each stage of the study is given in Fig. 1.

Although virtually all patients were aware about the beneficial effects of physical activity, main arguments against participation were (1) care of spouse (*n* = 3), (2) distance/travel time to the training center (*n* = 3), (3) fear not to meet the expectations of the training group (*n* = 1), and (4) unwillingness to accept randomization (*n* = 3). Five patients from the control group refused to repeat neuromuscular testing after 3 and 6 months, mainly due to discomfort of the investigations and/or the expenditure of time. Drop outs during the study period were due to personal reasons and an unrelated injury. The training attendance rate as a rough measure for the acceptance of the intervention was 84%.

Anthropometric data, information on prescribed medications, concomitant skeletal disorders, and details of previous fractures are summarized in Table 1. Routine laboratory work-up revealed normal findings (data not shown) and 25-hydroxyvitamin D_3_ serum concentration was below 75 nmol/l in five patients from the intervention group, and five from the control group.

**Table 1:** Characteristics of patients randomized to the intervention (INT) or control (CON) group at time of enrolment

	INT	CON	Δ_INT − CON_
Anthropometric data			
Female/male	11/2	13/1	−1
Age (years)	74 ± 3	76 ± 5	−2
Height (cm)	161 ± 8	159 ± 7	2
Weight (kg)	64 ± 11	67 ± 11	−3
BMI (kg m^−2^)	24 ± 3	26 ± 4	−2
Physical activity (self-reported) (h/week)	0.7 ± 0.6	0.8 ± 0.5	−0.1
*T*-score	−2.98 ± 0.61	−2.92 ± 1.09	−0.06
Skeletal disorders			
Arthritis	7	8	
Scoliosis	5	10	
Fractures of			
Distal radius	4	2	
Femoral neck	0	2	
Vertebrae	3	7	
Others	5	6	
Osteoporosis specific medication			
Bisphosphonates	9	6	
Vitamin-D supplementation	6	6	
Calcium	1	2	
Others	2	3	
Antihypertensive therapy			
Beta-blocker	5	1	
Others	4	6	
Analgesics	1	2	

Obesity, hypertension, a history of curatively treated oncological disease, and minor cardiac, renal or lung problems were noticed in four, seven, five, five, three and one patient from the intervention group, respectively, as well as in five, ten, one, five, one and two patients from the control group. Two patients per group suffered from type 2 diabetes mellitus.

### Neurophysiological investigations

All patients completed the neurophysiological investigations at baseline (Table 2). After 3 and 6 months data on neuromuscular function are available from 20 (nine controls) and 18 patients (nine per group), respectively.

**Table 2:** Resting peak twitch torques, evoked potentials, maximal and explosive voluntary strength, normalized muscle activity (RMS-EMG/*M*_max_) during iMVT and the initial phase of contraction at baseline (*t*_0_) for the training (INT) and the control group (CON)

Parameter	Pre		
	INT (*n* = 13)	CON (*n* = 14)	Δ INT − CON
Peak twitch torque (N m)			
Supramaximal single	12.2 ± 2.2	13.7 ± 2.8	−1.5
Supramaximal doublet	21.2 ± 3.5	23.6 ± 4.6	−2.4
H-reflex intensity	7.2 ± 2.7	6.7 ± 2.4	0.5
Evoked potentials			
*H*_max_ SOL (mV)	1.07 ± 0.18	1.73 ± 1.45	−0.66
*M*_max_ SOL (mV)	3.54 ± 1.76	4.54 ± 1.75	−1.00
*H*_max_/*M*_max_ SOL	0.37 ± 0.21	0.46 ± 0.33	−0.09
*M*_Hmax_/*M*_max_ SOL	0.17 ± 0.12	0.19 ± 0.12	−0.02
*M*_sup_ SOL (mV)	5.16 ± 2.09	5.07 ± 2.44	0.09
V wave SOL (mV)	0.49 ± 0.43	0.58 ± 0.24	−0.09
*V*/*M*_sup_ SOL	0.091 ± 0.064	0.150 ± 0.096	0.059
*M*_max_ MG (mV)	3.78 ± 1.58	2.99 ± 0.63	0.79
*M*_max_ LG (mV)	4.03 ± 1.27	3.26 ± 0.82	0.77
*M*_max_ TA (mV)	3.45 ± 1.16	2.95 ± 0.74	0.50
Isometric maximum voluntary torque (N m)	84.9 ± 15.6	81.0 ± 20.9	3.9
RMS-EMG_iMVT_/*M*_max_			
TS	0.046 ± 0.009	0.044 ± 0.008	0.002
TA	0.018 ± 0.004	0.020 ± 0.004	0.002
Rate of torque development (N m s^−1^)			
0–50 ms	82.0 ± 31.3	108.1 ± 56.7	−26.1
0–200 ms	170.4 ± 67.5	186.8 ± 95.3	−16.4
RMS-EMG_RTD_/*M*_max_			
TS 0–50 ms	0.032 ± 0.011	0.037 ± 0.010	−0.005
TS 0–200 ms	0.041 ± 0.012	0.041 ± 0.010	0.000
TA 0–50 ms	0.015 ± 0.004	0.018 ± 0.004	−0.003
TA 0–200 ms	0.017 ± 0.003	0.019 ± 0.004	−0.002

*Diff* difference between means, *H*_*max*_ maximal H-reflex, *M*_*max*_ maximal M-wave, *M*_*Hmax*_ submaximal M-wave evoked at H_max_ intensity, *H*_*sup*_ H-reflex during MVC, *M*_*sup*_ maximal M-wave during MVC, *M*_*Hsup*_ submaximal M-wave evoked at H_sup_ intensity during MVC, *SOL* soleus, *MG* medial gastrocnemius, *LG* lateral gastrocnemius, *TS* triceps surae, *TA* tibialis anterior

### Isometric maximum voluntary torque

**Fig. 3:**
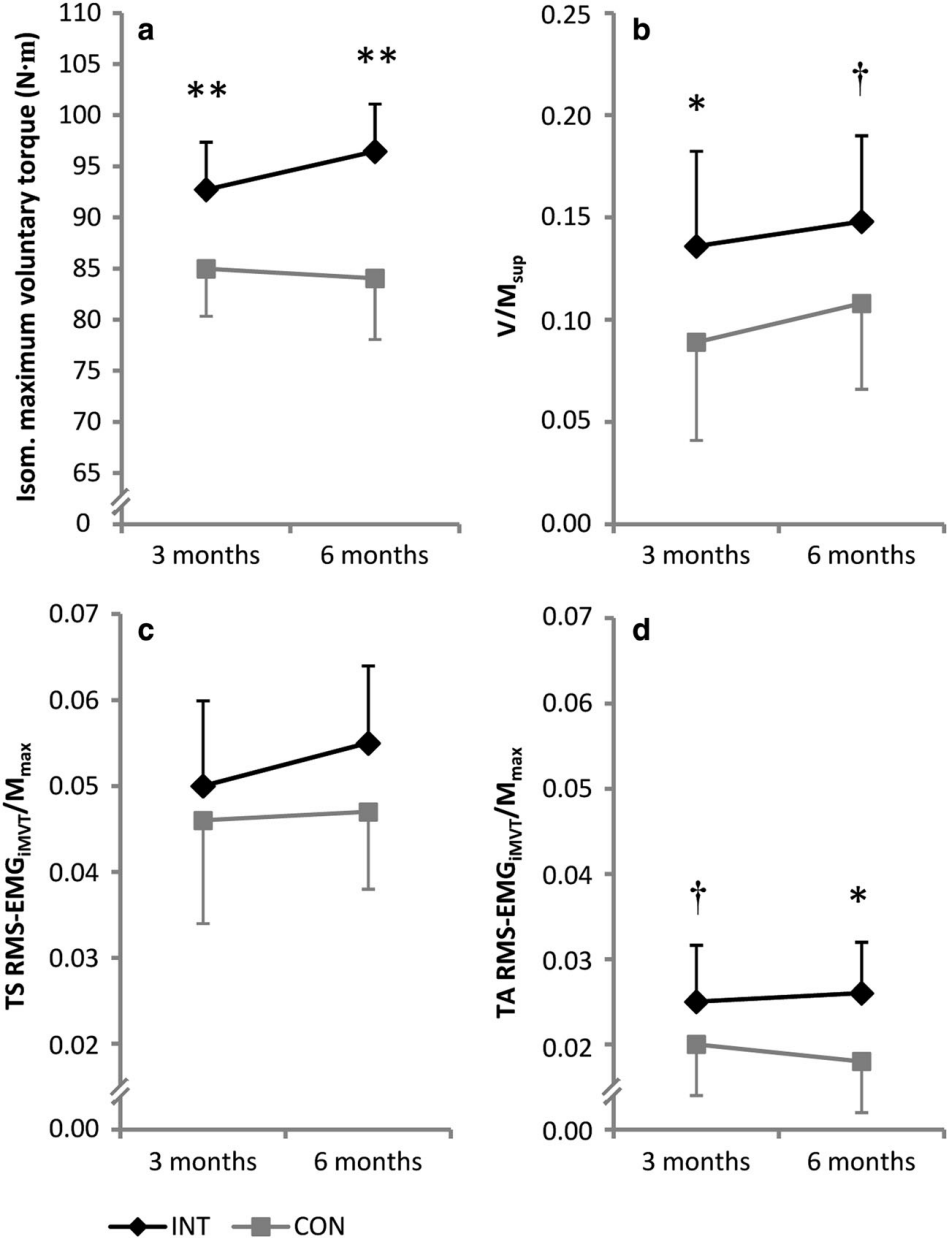
Effect of the training intervention (INT) on isometric maximum voluntary torque (**a**), normalized V-wave (*V*/*M*_sup_, **b**) and normalized muscle activity during iMVT (RMS-EMG_iMVT_/*M*_max_, **c**, **d**). *Denotes a significant difference between groups (*P ≤ 0.05; **P ≤ 0.01) and ^**†**^denotes a statistical tendency towards a significant difference between groups (*P* *≤* 0.10). *CON* control group, *TS* triceps surae, *TA* tibialis anterior

After 3-months training period, iMVT was significantly higher by 7.7 N m (3.3–12.2 N m, *P* < 0.01) for the intervention group compared to the controls. After 6 months of training, the difference between groups in iMVT was 12.4 N m (6.4–18.5 N m, *P* < 0.01) (Fig. 3a). At these time points, also the normalized V-wave of soleus (*V*/*M*_sup_) differed between both groups [3 months: 0.047 (0.000–0.093, *P* < 0.05); 6 months: 0.041 (−0.001 to 0.083), *P* = 0.06] (Fig. 3b). The intervention had no effect on normalized muscle activity of the triceps surae (TS RMS-EMG_iMVT_/M_max_) (Fig. 3c). We observed an increased normalized muscle activity of tibialis anterior during MVC already after 3 months of training [0.005 (−0.001 to 0.010, *P* = 0.10)] (Fig. 3d). Nevertheless, we do not know whether or not this was due to the training itself. After 6 months of training, activity of the tibialis anterior muscle was significantly different between groups [0.007 (0.000–0.015, *P* < 0.05)].

### Rate of torque development

**Fig. 4:**
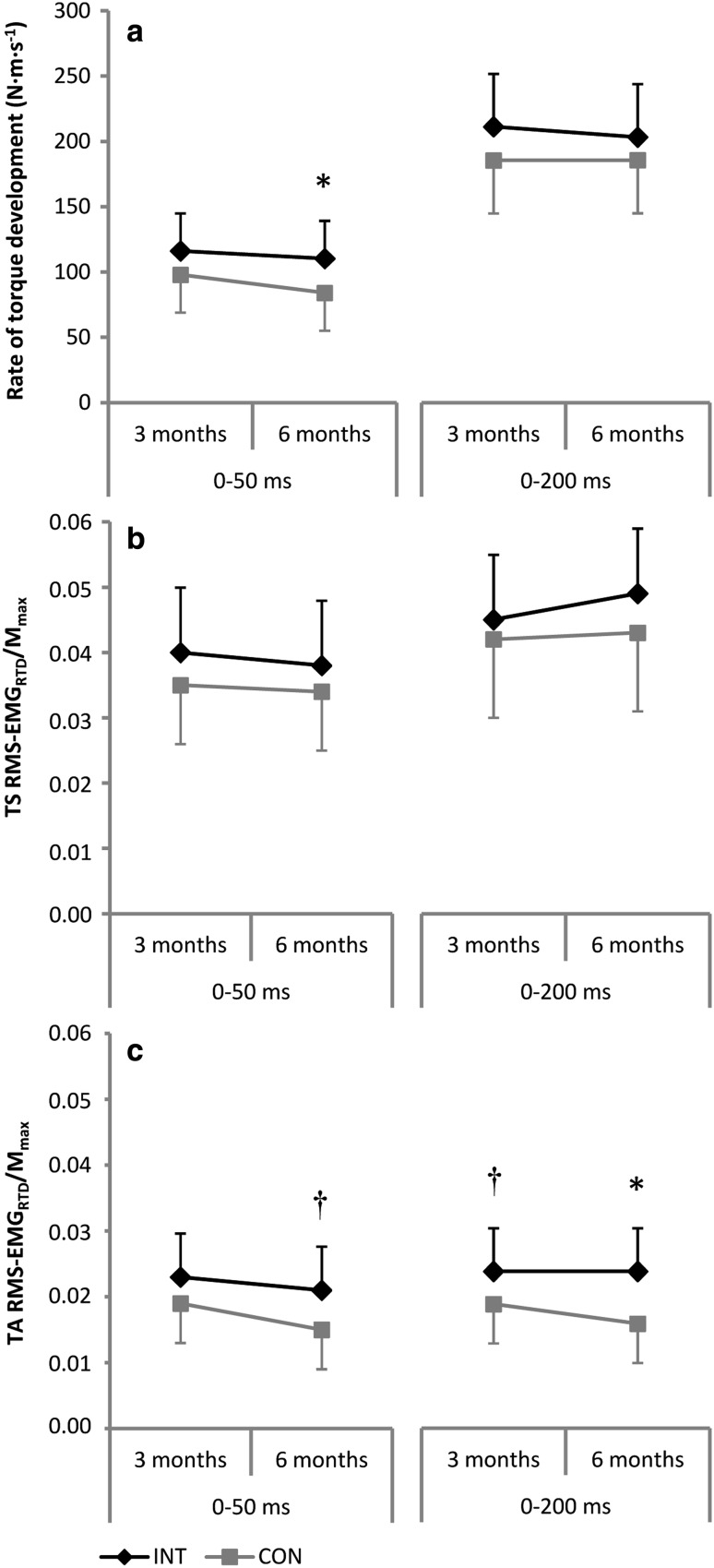
Effect of the training intervention (INT) on rate of torque development (**a**), normalized muscle activity of the triceps surae (TS RMS-EMG_RTD_/*M*_max_, **b**) and normalized muscle activity of the tibialis anterior muscle (TA RMS-EMG_RTD_/*M*_max_, **c**) in the time intervals 0–50 and 0–200 ms. ^**†**^Denotes a statistical tendency towards a significant difference between groups (*P* ≤ 0.10). *CON* control group, *TS* triceps surae, *TA* tibialis anterior

No significant difference between groups in RTD 0–50 ms and RTD 0–200 ms were observed after 3 months of training (Fig. 4a). In the initial phase of triceps surae contraction, i.e., 0–50 and 0–200 ms, muscle activity was similar in both groups after 3 months of training (Fig. 4b). Likewise, activity of the tibialis anterior muscle in the time interval 0–50 ms was similar in both groups. Activity of the tibialis anterior muscle in the time interval 0–200 ms was higher for the intervention group after 3 months of training [0.005 (−0.001 to 0.010, *P* = 0.08)] (Fig. 4c). However, we cannot rule out that this was by chance rather than being an effect of the training. After 6 months of training, a significant difference between groups was found for RTD 0–50 ms [26.3 N m s^−1^ (1.2–51.5 N m s^−1^, *P* < 0.05)], whereas RTD 0–200 ms remained unchanged (Fig. 4a). In the initial phase of triceps surae contraction, i.e., 0–50 and 0–200 ms, muscle activity was similar in both groups after 6 months of training (Fig. 4b). Activation of the tibialis anterior muscle in the time interval 0–50 ms was higher for the intervention group after 6 months of training [0.006 (−0.001 to 0.013, *P* = 0.09)] and activation of this muscle in the time interval 0–200 ms differed significantly between groups at this particular point of time [0.007 (0.001–0.014, *P* < 0.05)] (Fig. 4c).

### Twitch torque parameters

Twitch mechanical parameters changed with training, i.e., peak twitch torque induced by single and doublet stimulation was higher in the intervention group compared to controls after 3 and 6 months of training (Table 3).

**Table 3:** Resting peak twitch torques and evoked potentials after 3 and 6 months of training for the intervention (INT) and the control group (CON)

Parameter	3 months				6 months			
	INT (*n* = 11)	CON (*n* = 9)	Diff. (95% CI), Δ INT − CON	*P*	INT (*n* = 9)	CON (*n* = 9)	Diff. (95% CI), Δ INT − CON	*P*
Peak twitch torque (N m)								
Supramaximal single	14.3 ± 1.0	13.2 ± 1.0	1.1 (0.2–2.1)	0.03*	14.9 ± 1.0	13.6 ± 1.0	1.3 (0.4–2.4)	0.01*
Supramaximal doublet	25.1 ± 2.2	23.0 ± 2.2	2.1 (−0.1 to 4.2)	0.06†	25.5 ± 1.8	23.5 ± 1.8	2.0 (0.2–3.8)	0.04*
H-reflex intensity	6.4 ± 1.65	7.8 ± 1.65	−1.4 (−3.5 to 0.8)	0.20	7.0 ± 2.1	8.9 ± 2.1	−1.9 (−4.1 to 0.3)	0.09**†**
Evoked potentials								
*H*_max_ SOL (mV)	1.29 ± 0.46	1.31 ± 0.46	−0.02 (−0.64 to 0.60)	0.94	1.47 ± 0.79	1.55 ± 0.79	−0.08 (−0.94 to 0.78)	0.85
*M*_max_ SOL (mV)	3.89 ± 0.89	4.06 ± 0.89	−0.17 (−1.04 to 0.70)	0.68	3.74 ± 1.01	3.55 ± 1.01	0−19 (−0.84 to 1.23)	0.70
*H*_max_/*M*_max_ SOL	0.34 ± 0.12	0.36 ± 0.12	−0.02 (−0.18 to 0.14)	0.79	0.36 ± 0.22	0.44 ± 0.22	−0.08 (−0.31 to 0.15)	0.46
*M*_Hmax_/*M*_max_ SOL	0.19 ± 0.14	0.14 ± 0.14	0.05 (−0.14 to 0.24)	0.58	0.21 ± 0.14	0.22 ± 0.14	−0.01 (−0.16 to 0.15)	0.91
*M*_sup_ SOL (mV)	4.89 ± 1.15	5.74 ± 1.15	−0.85 (−1.95 to 0.25)	0.12	4.89 ± 1.59	5.66 ± 1.59	−0.77 (−2.38 to 0.84)	0.32
V wave SOL (mV)	0.65 ± 0.26	0.51 ± 0.26	0.14 (−0.11 to 0.39)	0.24	0.73 ± 0.20	0.57 ± 0.20	0.16 (−0.04 to 0.36)	0.11
*M*_max_ MG (mV)	3.38 ± 0.91	3.46 ± 0.91	−0.08 (−0.97 to 0.80)	0.85	3.52 ± 1.07	3.48 ± 1.07	0.04 (−1.04 to 1.13)	0.93
*M*_max_ LG (mV)	3.71 ± 1.05	3.40 ± 1.05	0.31 (−0.73 to 1.35)	0.54	3.20 ± 0.89	3.87 ± 0.89	−0.67 (−1.60 to 0.26)	0.14
*M*_max_ TA (mV)	3.21 ± 0.88	3.70 ± 0.88	−0.49 (−1.35 to 0.36)	0.24	2.77 ± 1.03	3.39 ± 1.03	−0.62 (−1.67 to 0.42)	0.22

*Diff. (95% CI)* difference between means (95% confidence interval), *H*_*max*_ maximal H-reflex, *M*_*max*_ maximal M-wave, *M*_*Hmax*_ submaximal M-wave evoked at *H*_max_ intensity, *H*_*sup*_ H-reflex during MVC, *M*_*sup*_ maximal M-wave during MVC, *M*_*Hsup*_ submaximal M-wave evoked at H_sup_ intensity during MVC, *SOL* soleus, *MG* medial gastrocnemius, *LG* lateral gastrocnemius, *TA* tibialis anterior. Data are baseline-adjusted means ± baseline-adjusted standard deviation. *Denotes a significant difference between groups (*P* ≤ 0.05) and ^**†**^denotes a statistical tendency towards a significant difference between groups (*P* ≤ 0.10)

## Discussion

We hypothesized that step aerobics training might be suitable to preserve or even improve the musculoskeletal unit in older patients with osteoporosis and find this issue worthwhile to be investigated in a randomized trial. However, this is challenging due to the age of the target group and the type of intervention desired. In fact, recruitment of patients was challenging and reasonable resources are required for training of even such a small number of patients. On the one hand, it was greatly appreciated and supportive for the protocol adherence of our participants, on the other hand this is probably not feasible if the number of patients is increased.

We investigated the effects on neuromuscular function and decided for examination of the plantar flexors instead of the quadriceps muscles. Although the latter are equally important for step aerobic training, stimulation of the femoral nerve is less comfortable than stimulation of the posterior tibial nerve. However, about one third of the patients from the control group refused to repeat this type of examinations secondary to the associated discomfort and/or the expenditure of time (2 h for the complete investigation per patient). Due to financial restrictions, we were not able to offer the participants financial compensation.

We have clearly shown that maximal voluntary strength as well as neural activation of soleus (*V*/*M*_sup_) were improved after 3 and 6 months of training compared to controls. Six months of training significantly improved explosive voluntary strength in the time interval 0–50 ms. In addition, differences between groups were noted with respect to the evoked peak twitch torques.

To the best of our knowledge, this is the first study analyzing the effects of modified step aerobics training on maximal voluntary strength, V-wave and twitch mechanical parameters in older patients suffering from osteoporosis. Most studies on physical adaptations following step aerobics training focussed on changes in functional fitness [16, 34, 35] and balance performance [36]. Although it has been shown that step aerobics has the potential to increase voluntary strength [16, 35], little is known about the underlying neuromuscular adaptations. Our data indicate that step aerobics training increased maximal voluntary strength of the plantar flexors in osteoporotic patients due to an increased neural drive to the agonistic muscle and improved contractile function of the triceps surae muscle. The strength gain observed after 3 months of training were probably due to neural and muscular adaptations, i.e., an increased normalized V-wave (*V*/*M*_sup_) and peak twitch torque. By contrast, the strength gain obtained during the subsequent 3 months of training was probably mainly due to muscular changes because indices of neural activation of muscles remained at the same level while peak twitch torque of the plantar flexors continued to rise.

An enhanced normalized V-wave was previously observed after strength training in young adults [32] and probably reflects an increased α-motoneuron firing frequency and/or recruitment. In this context, it has to be noted that the V-wave response is susceptible to alterations in presynaptic inhibition of Ia afferents as well [32]. It has been suggested that voluntary strength increases during the first weeks of training are primarily due to increased muscle activity and subsequent hypertrophy of muscle fibers [37]. Interestingly, co-contraction of tibialis anterior during MVC was different between groups. Because changes in co-contraction of tibialis anterior during plantar flexion did not seem to enhance reciprocal inhibition of the triceps surae [38], V-wave responses were probably not affected by the increased muscle activity. In general, strength training of an agonistic muscle decreases co-activation of the antagonist [37]. Our patients had to produce muscular forces to descend and ascend a stepper during the training. This exercise is not only characterized by high moments acting on the joint [39] but also requires to keep balance. This can be achieved by increasing tibialis anterior muscle activity during torque production of the plantar flexors because an increased activity of antagonistic muscles at the same time induces opposing muscle forces that increase the stiffness about the involved joint. Furthermore, activation of the dorsal flexors is required during stepping upstairs to avoid collision of the tiptoes with the stepper.

The training regimen induced changes in contractile properties of the plantar flexors, i.e., an increase in the twitch mechanical parameters induced by electrical stimulation at rest. It has been shown that peak twitch torque of the plantar flexors induced by electrical stimulation of the posterior tibial nerve is highly correlated with triceps surae cross-sectional area assessed by peripheral quantitative computer tomography [40]. Thus, our findings point to training-related hypertrophic changes in the triceps surae muscle even in these older osteoporotic patients.

Explosive voluntary strength in the time interval 0–50 ms was increased following 6 months of training, whereas normalized agonistic muscle activity has not changed. Most likely, adaptations at the muscular level were responsible for the enhanced mechanical output [41]. In addition, it has been shown that voluntary RTD is positively related to the stiffness of the tendon-aponeurosis complex [42]. Therefore, it may be that changes in stiffness of the tendon-aponeurosis complex following the intervention contributed to the observed result.

During ascending and descending stairs moments at weight-bearing joints are much greater than those during level walking [39]. These high moments have to be generated by the muscles of the lower limbs and may stimulate neural and muscular adaptations comparable to those induced by strength training. The training-related neuromuscular adaptations of the plantar flexors in our patients are well in line with these findings. Thus, modified step aerobics appears to be not only a feasible training regimen but also to enhance neuromuscular performance even in older osteoporotic patients. It remains to be elucidated whether the training regimen will also reduce the risk of falls and/or low-energy fractures.

Our study has some limitations. First of all, our sample might be biased as we invited patients from one center only and those who finally agreed are certainly those most interested in an active contribution to maintain skeletal health and were in a rather good physical condition. Although we failed to prospectively announce the study at a public registry, one may doubt that this would have helped to recruit patients. Second, randomization was according to age and sex, whereas duration of disease, nutritional behavior, osteoporosis specific medication, neuromuscular performance and comorbidities were not considered at all. Instead, we wanted to learn about the conditions and specific considerations inherent to a physical intervention in old and perhaps already disabled patients with osteoporosis. Such specific considerations are for example related to (1) recruitment and randomization of elderly patients with osteoporosis, (2) type, timing (i.e., morning or evening), frequency, duration per session and intensity of the intervention, (3) the accessibility of the training center by public transportation, (4) the number of trainers and supportive staff required during exercise and data acquisition at time of study examinations, and (5) the need for heart rate monitoring during the training session.

All participants of the intervention group were really eager not to miss the training sessions and roughly half of the intervention group continued the training for additional 6 months. Furthermore, at the end of the study period even half of the control group wanted to start the same training protocol.

## Conclusion

In conclusion, our intervention was well accepted and is worth to be evaluated within the setting of a large randomized and ideally multicenter clinical trial. This should allow not only to control for comorbidities and disease-specific medications, but also for thorough investigation of efficacy regarding preservation and even improvement of musculoskeletal health and mobility. However, to investigate the training program as described would hardly be feasible in a large randomized clinical trial and would cause significant financial expenses. It remains to be elucidated whether this is balanced by reduction of expenditures for medication and hospitalization.

## Abbreviations

BMD: Bone mineral density
CON: Control group
DXA: Dual-energy X-ray absorptiometry
*H*_max_: Maximal *H*-reflex
HRpQCT: High resolution peripheral quantitative computer tomography
iMVT: Isometric maximum voluntary torque
INT: Intervention group
ISI: Interstimulus intervals
*M*_Hmax_: Submaximal M-wave evoked at *H*_max_ intensity
*M*_max_: Maximal M-wave
*M*_sup_: Maximal M-wave during MVC
MVC: Maximum voluntary contraction
RMS-EMG: Root mean square of the EMG signal
RTD: Rate of torque development
